# A new Cretaceous genus of xyelydid sawfly illuminating nygmata evolution in Hymenoptera

**DOI:** 10.1186/1471-2148-14-131

**Published:** 2014-06-17

**Authors:** Mei Wang, Alexandr P Rasnitsyn, Chungkun Shih, Dong Ren

**Affiliations:** 1 College of Life Sciences, Capital Normal University, 105 Xisanhuanbeilu, Haidian District, Beijing 100048, China; 2 Palaeontological Institute, Russian Academy of Sciences, 123, Profsoyuznaya ul, Moscow 117997, Russia; 3 Department of Palaeontology, Natural History Museum, Cromwell Road, London SW7 5BD, UK

**Keywords:** *Rectilyda*, Xyelydidae, Symphyta, Yixian formation, Nygmata, China

## Abstract

**Background:**

Nygmata are prominent glandular structures on the wings of insects. They have been documented in some extant insects, including several families of Neuroptera and Mecoptera, the majority of Trichoptera, and a few of the hymenopteran Symphyta. However, because nygmata are rarely preserved in compression fossils, their early development and evolution are still enigmatic. For example, the only documented nygmata in the Hymenoptera are on the forewings of the Triassic xyelids *Asioxyela paurura* and *Madygenius primitives.*

**Results:**

This study describes and illustrates a new genus and species from the family Xyelydidae, *Rectilyda sticta* gen. et sp. nov., from the Early Cretaceous Yixian Formation of Duolun County, Inner Mongolia, China. This genus has 1-RS reclival and linearly aligned with 1-M, which is different from all other genera in the Xyelydidae. In addition, *R. sticta* gen. et sp. nov. has clearly preserved nygmata: four symmetrical nygmata on each forewing and two on each hind wing.

**Conclusion:**

Previous reports of nygmata on the forewings of Triassic xyelids and extant sawflies, together with this new fossil record of nygmata, provide rare insights into their developmental trends, as well as into the evolution of hymenopterans and insects in general.

## Background

Xyelydidae is an extinct family known from compression fossils ranging in age from the later Early (or earlier Middle) Jurassic to Early Cretaceous. This family, considered to be the most primitive of the Pamphilioidea, plays an important role as an ancestral group in the evolution of the superfamily [1-4]. There are currently 18 species in seven genera recognized within this family [3,5-10], which are summarized in Table 1.

**Table 1:** Fossil record of Xyelydidae

**Taxa**	**Locality and horizon**	**Wing length (mm)**
*Ferganolyda cubitalis* Rasnitsyn, 1983	Sai-Sagul, Fergana, Kyrgyzstan; Lower or Middle Jurassic Sogul Formation	15
*F. radialis* Rasnitsyn, 1983	Sai-Sagul, Fergana, Kyrgyzstan; Lower or Middle Jurassic Sogul Formation	11
*F. sogdiana* Rasnitsyn, 1983	Sai-Sagul, Fergana, Kyrgyzstan; Lower or Middle Jurassic Sogul Formation	16
*F. scylla* Rasnitsyn, Zhang & Wang, 2006	Daohugou, Inner Mongolia, China; Middle Jurassic Jiulongshan Formation	8 as preserved
*F. charybdis* Rasnitsyn, Zhang & Wang, 2006	Daohugou, Inner Mongolia, China; Middle Jurassic Jiulongshan Formation	16.6
*F. chungkuei* Rasnitsyn, Zhang & Wang, 2006	Daohugou, Inner Mongolia, China; Middle Jurassic Jiulongshan Formation	15.4 (male) 9.0 (female)
*Mesolyda jurassica* Rasnitsyn, 1963	Mikhailovka, Karatau, southern Kazakhstan; Upper Jurassic Karabastau Formation	9
*M. sibirica* Rasnitsyn, 1983	Uda, Buryat Republic, Siberia; Upper Jurassic Uda Formation	13.5
*Novalyda cretacica* Gao, Engel, Shih & Ren, 2013	Huangbanjigou, Liaoning, China; Lower Cretaceous Yixian Formation	6.83
*Prolyda karatavica* Rasnitsyn, 1968	Mikhailovka, Karatau, southern Kazakhstan; Upper Jurassic Karabastau Formation	6
*P. depressa* Rasnitsyn, 1969	Mikhailovka, Karatau, southern Kazakhstan; Upper Jurassic Karabastau Formation	?
*P. xyelocera* Rasnitsyn, 1968	Mikhailovka, Karatau, southern Kazakhstan; Upper Jurassic Karabastau Formation	5
*Rectilyda sticta* gen. et sp. nov.	Nanyingpan, Inner Mongolia, China; Lower Cretaceous Yixian Formation	17.5
*Strophandria grossa* Rasnitsyn, 1968	Mikhailovka, Karatau, southern Kazakhstan; Upper Jurassic Karabastau Formation	15
*S. moderata* Rasnitsyn, 1983	Mikhailovka, Karatau, southern Kazakhstan; Upper Jurassic Karabastau Formation	9
*Sagulyda arcuata* Rasnitsyn, 1983	Sai-Sagul, Fergana, Kyrgyzstan; Lower or Middle Jurassic Sogul Formation	10
*S. ferganica* Rasnitsyn, 1983	Sai-Sagul, Fergana, Kyrgyzstan; Lower or Middle Jurassic Sogul Formation	12
*S. magna* Rasnitsyn, 1983	Sai-Sagul, Fergana, Kyrgyzstan; Lower or Middle Jurassic Sogul Formation	ca. 30
*Xyelyda excellens* Rasnitsyn, 1968	Mikhailovka, Karatau, southern Kazakhstan; Upper Jurassic Karabastau Formation	7

Very few Xyelydidae fossils have been reported from China and only three species have been described from the Jiulongshan Formation of Daohugou, Inner Mongolia, China [8]: *Ferganolyda* Rasnitsyn, 1983, *F. scylla* Rasnitsyn, Zhang & Wang, 2006; *F. charybdis* Rasnitsyn, Zhang & Wang, 2006; and *F. chungkuei* Rasnitsyn, Zhang & Wang, 2006. In addition, one species from the Yixian Formation of Huangbanjigou, Liaoning, China has been described: *Novalyda cretacica* Gao, Engel, Shih & Ren, 2013. *Novalyda cretacica* is the only record reported from the Early Cretaceous, representing the youngest record of this family to date [10].

In this study, we describe *Rectilyda sticta* gen. et sp. nov. from the Yixian Formation of Nanyingpan Village, the Sandaogou Township, Duolun County, Inner Mongolia, China. Based on a unique combination of primitive and more derived characters of this sawfly, we present a new genus and assign it to the family Xyelydidae. This insect fauna lived about 126 Mya, and belonged to the famous Jehol biota of the Early Cretaceous [11-14]. According to a survey of the CNUB insect fossil collection, Duolun has yielded many insect fossils, including bugs [15], basal fleas [16], and rare specimens of sawflies. The only sawfly that has been described so far is *Hoplitolyda duolunica* Gao, Shih, Rasnitsyn & Ren, 2013, which belongs to Praesiricidae of the superfamily Pamphilioidea. It represents the largest fossil hymenopteran to date, with an estimated body length of 55.0 mm and wing span of 92.0 mm [17]. *Rectilyda sticta* gen. et sp. nov. is, therefore, the second sawfly described from the Duolun locality.

## Methods

The specimen was examined and then photographed, either dry or wetted with 95% ethanol, using a Leica MZ 16.5 dissecting microscope (Leica, Wetzlar, Germany) with a Leica DFC500 digital camera attached. The line drawings were prepared using Adobe Illustrator CS2 and Adobe Photoshop CS5 software. The wing venation nomenclature used in this article was modified after Rasnitsyn [1,2]. The type material described is deposited in the Key Lab of Insect Evolution and Environmental Changes, College of Life Sciences, Capital Normal University, in Beijing, China (CNUB; Ren Dong, Curator).

No specific permits were required for collection of the insect fossils, including the materials from Nanyingpan Village, Sandaogou Township, Duolun County, Inner Mongolia, China.

## Results

### Description of the specimens

Hymenoptera Linnaeus, 1758.

Pamphilioidea Cameron, 1890.

Xyelydidae Rasnitsyn, 1968.

*Rectilyda* Wang, Rasnitsyn, Shih & Ren, gen. nov.

#### Etymology

The generic name is a combination of the Latin “Rect-”, meaning straight (referring to the alignment of 1-RS and 1-M), and *Lyda*, a junior synonym of *Pamphilius* Latreille, 1802, often used as a suffix for generic names in Pamphilioidea. Gender feminine.

#### Diagnosis

Antenna with about 17 segments; the third antennal segment shorter than remaining segments combined; flagellomeres nearly as long as wide, becoming narrower and shorter towards the apex. Mandibles strong, with at least one small inner tooth. Forewing with SC bifurcate; pterostigma narrow and long, sclerotized completely; 1-RS reclival, as long as 1-M and linearly aligned with 1-M; 1-M nearly equal to 2-M in length; cell 3r at least 1.5 times as long as cells 1r and 2r. Hind wing with SC1 and SC2 present; crossvein m-cu distal to middle of cell rm; cu-a before middle of cell mcu.

#### Remarks

We assign *Rectilyda* gen. nov. to Xyelydidae mainly based on three primitive characters. This recognition is by necessity, as the family is paraphyletic and reclassification into one or several monophyletic parts is complicated under the current state of knowledge [4,7]. The primitive characters are: 1) SC bifurcate in the forewing; 2) M + Cu smoothly bent; 3) cell 1mcu relatively large and 1cu-a located distad middle of cell 1mcu. *Rectilyda* differs from other members of Xyelydidae in having 1-RS aligned with, and as long as, 1-M and the third antennal segment shorter than remaining segments combined. Other xyelydids always have 1-RS proclival, distinctly angled with 1-M and much shorter than 1-M; 1-RS is rarely subequal to 1-M, and the third antennal segment is relatively longer and thicker than the subsequent ones combined.

*Rectilyda sticta* Wang, Rasnitsyn, Shih & Ren, sp. nov.

#### Diagnosis

As for the genus by monotypy.

#### Etymology

The specific name is derived from the Greek word “stictos” meaning spotted or punctured, referring to the nygmata on the wings.

#### Holotype

CNU-HYM-LB-2012125, deposited in CNUB, sex unknown, a well-preserved sawfly, with an almost complete body, including parts of the antennae, both forewings, hind wings, and parts of the legs.

#### Locality and horizon

Nanyingpan Village, Sandaogou Township, Duolun County, Inner Mongolia, China. Yixian Formation, Early Cretaceous.

#### Description

**Figure 1:**
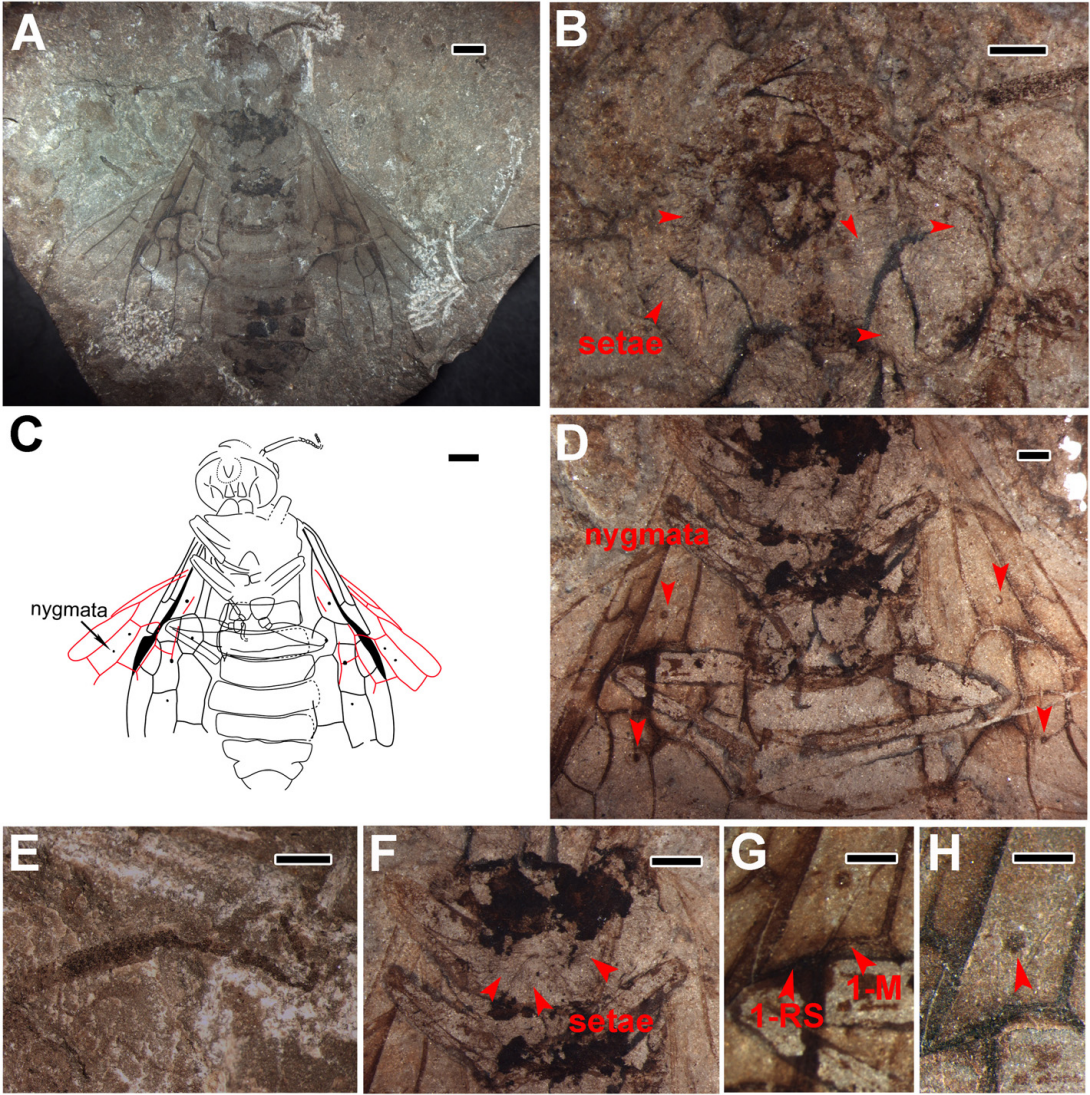
***Rectilyda sticta *gen. et sp. nov.** Holotype, CNU-HYM-LB2012125. **A**, Habitus. **B**, Head (with addition of alcohol). **C**, Line drawing. **D**, Part of legs (with addition of alcohol). **E**, Part of antennae (with addition of alcohol). **F**, Setae on mesothorax. **G**, Part of left forewing with 1-RS aligned with 1-M. **H**, nygma on cell 1rm. Scale bars: 2 mm in **A** and **C**; 0.5 mm in **B**, **D**, **E**, **F** and **G**; 0.2 mm in **H**.

Color not reliable because of absence of counterpart fossil; as preserved, body infuscate, with mouthparts, antennae, part of mesothorax and abdominal segments 4–8 darker; forewing somewhat infuscate sub-basally, with pterostigma dark, especially in costal area along veins (Figure 1A). Entire body and all legs (excluding tarsi) with dense, long, dark setae.

**Figure 2:**
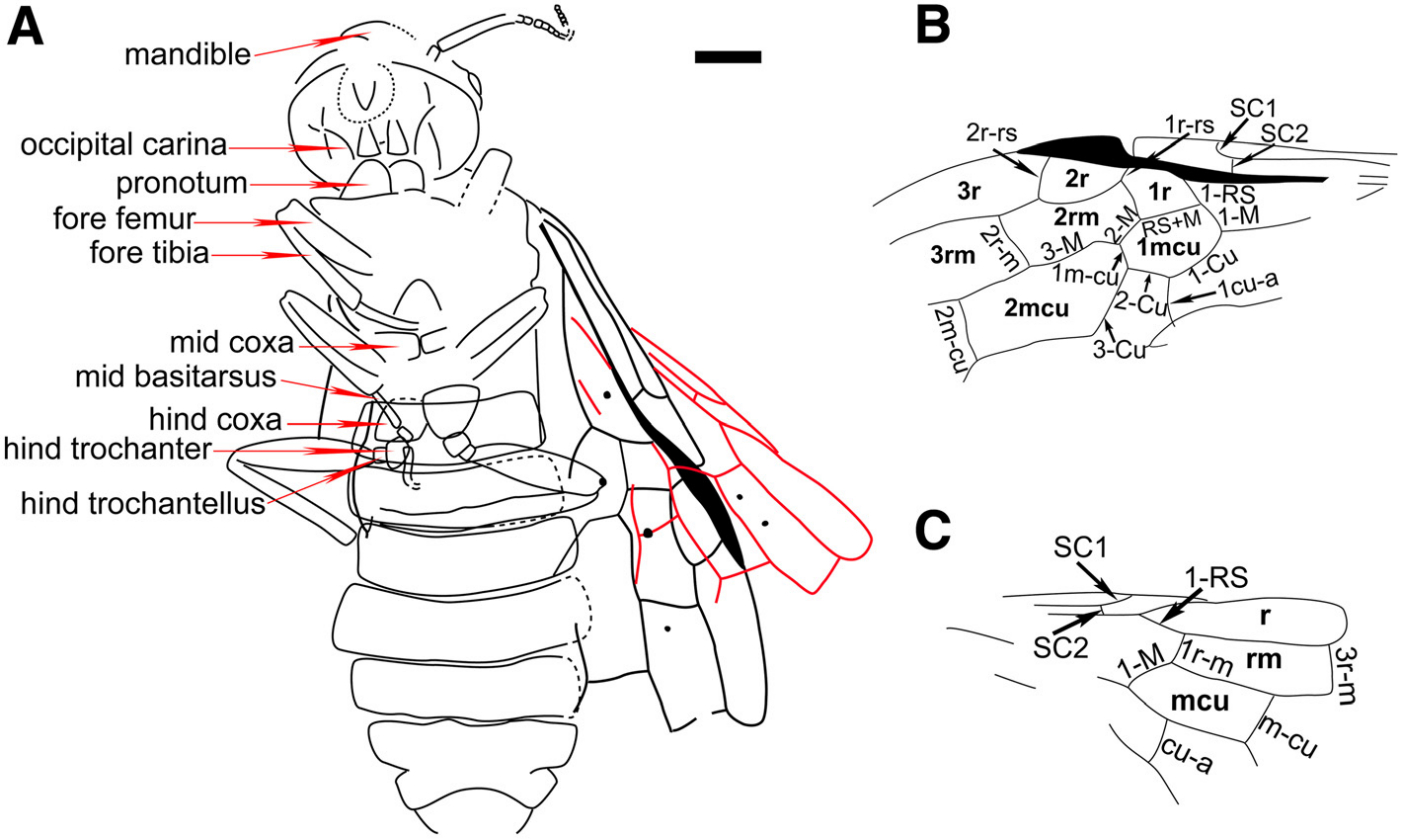
**Line drawings of *Rectilyda sticta *gen.et sp. nov.** Holotype. **A**, Body. **B**, Left forewing. **C**, Right hind wing. Scale bars: 2 mm.

**Figure 3:**
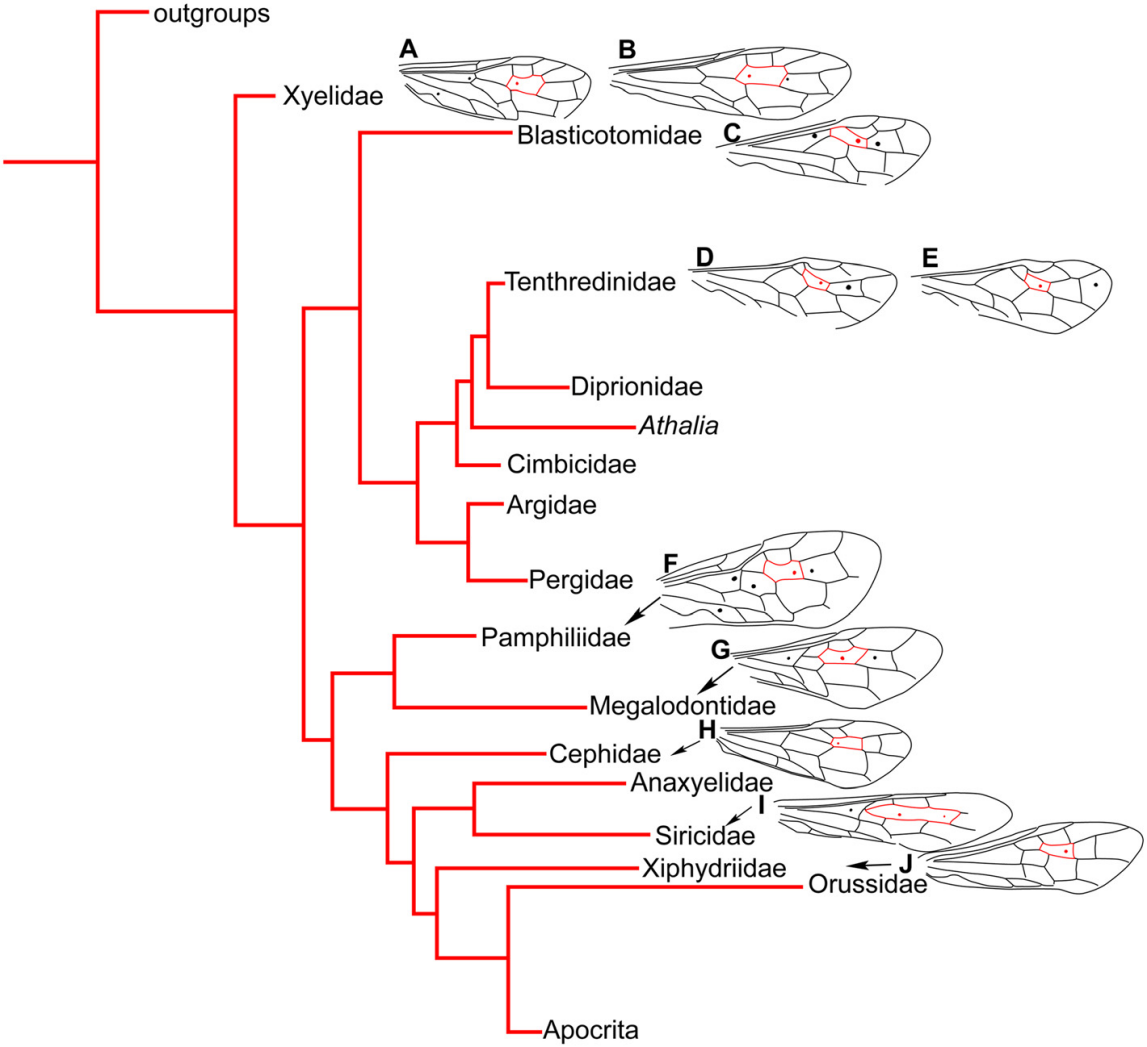
**Cladogram of extant basal Hymenoptera after Ronquist et al. (2012, Figure **3**, slightly modified), with relevant forewing venation containing nygmata among extant taxa mapped onto figure. A**, *Macroxyela ferruginea* Say, 1824 (Xyelidae). **B**, *Megaxyela major* Cresson, 1880 (Xyelidae). **C**, *Blasticotoma filiceti* Klug, 1834 (Blasticotomidae). **D**, *Empria candidata* Fallén, 1808 (Tenthredinidae). **E**, *Empria formosana* Prous & Heidemaa, 2012 (Tenthredinidae). **F**, *Onycholyda amplecta* Fabricius, 1804 (Pamphiliidae). **G**, *Megalodontes cephalotes* Fabricius, 1781 (Megalodontidae). **H**, *Cephus pygmeus* Linné, 1767 (Cephidae). **I**, *Tremex columba* Linné, 1763 (Siricidae). **J**, *Xiphydria camelus* Linné, 1758 (Xiphydriidae). Wing drawings based on images referred to in Table 2.

Head: relatively large, transverse, ovate, about 1.23 times as wide as long, nearly as wide as mesothorax (0.9 times), with eyes and ocelli indistinguishable; mandibles small, sickle-shaped, with at least one inner tooth; other detailed structures of mouthparts obscured (Figure 1B). Antennae with about 17 segments (including pedicel), pedicel as wide as 3^rd^ segment, the 3^rd^ one (1.45 mm in length) shorter than remaining segments combined (3.3 mm in length), thickest point of 3^rd^ segment 0.46 mm wide, 4^th^ segment 0.21 mm wide, apical flagellomere 0.09 mm wide; flagellomeres gradually shorter and narrower towards apex, each flagellomere becoming flat and subquadrate (Figure 1E).Thorax: slightly wider than head, anterior edge of mesoscutum nearly straight (Figure 2A), mesothorax covered with some short setae (Figure 1F).Legs (Figures 1D and 2A): coxae trapezoidal (hind coxae 0.87 times as long as wide); two trochanters in hind legs, rectangular, covered with dense bristles, proximal one about 1.82 times as long as and 1.42 times as wide as distal one. Femora fusiform, about 3.5 times as wide as long; hind femora shorter than mesothorax; fore and mid femora narrower than hind femora. Tibiae comparatively narrow and long; hind tibiae longer than fore and mid tibiae and about 1.2 times as long as hind femora. Tarsi incompletely preserved, mid claw apparently with small submedial tooth.Wing: (Figure 2B) with SC bifurcate, SC1 bent gently upward and intersecting C; SC2 short, meeting R slightly before SC1 and meeting C, subperpendicular to R; R nearly straight, gently bent anteriorly in distal half, thickened before pterostigma; pterostigma narrow (0.57 mm wide), sclerotized completely. 1-RS reclival, inclined towards wing base, aligned with and meeting 1-M, about equal in length (Figure 1G). R between SC2 and 1-RS about 0.7 times as long as 1-RS and 1-M combined; 1r-rs inclined, 0.34 times as long as 2r-rs; 2r-rs bent towards wing apex; M + Cu bent smoothly; 2r-m inclined towards wing base and separated from 2r-rs by 0.7 times of its own length, located distad middle of cell 2mcu; 3r-m separated from apex of cell 3r by 0.67 of its length; 2r-m as long as 3r-m; 2-M nearly equal to 1-M; 1 m-cu 0.87 times as long as 2-M and about 0.57 times as long as 2-Cu; 2-M and 3-M meeting at distinct obtuse angle; 1cu-a bent distinctly towards wing apex, nearly as long as 2-Cu and placed apparently distad middle of cell 1mcu; 2 m-cu curved, almost at middle of cell 3rm; cell 2rm almost equal to 3 rm in length, and 0.75 times as long as and 0.73 times as wide as cell 2mcu. Four symmetrical nygmata present (Figure 1C) in cells 1rm (Figure 1D and H), 1mcu, 2rm (Figure 1D) and 3rm. In right hind wing (Figure 2C), vein SC well-developed with two branches. Cell r widely rounded apically; 1-RS slightly longer than 1-M; crossvein 1r-m distant from bases of both RS and M, about 0.65 times as long as 1-RS; 3r-m near apex of cell r, crossvein m-cu long, joining 2-M distal of midlength of cell rm, separated from 3r-m by nearly its length. Crossvein cu-a slightly curved towards wing apex, proximal to midlength of cell mcu. Two symmetrical nygmata in cell rm (Figure 1C).

Abdomen: eight abdominal terga visible, fourth tergum slightly longer than other segments. Genitalia indistinct (sex unknown).

#### Dimensions of holotype (in mm)

CNU-HYM-LB-2012125: Body length (excluding antennae) 24.0, head length without mandible 4.9, width 6.1, forewing length up to end of cell 3r 17.5, as preserved (full length, ca. 20.0).

## Discussion

### Unique characters in *Rectilyda sticta* gen. et sp. nov

Since most xyelydids have been described from detached wings without preserved bodies, we summarize the reported data of wing lengths for all species of xyelydids in Table 1. The data show that forewing lengths (up to the end of cell 3r) vary substantially from 5 mm in *Prolyda xyelocera* Rasnitsyn, 1968 to 17.5 mm in *R. sticta* gen. et sp. nov. The data show that *R. sticta* is the second largest xyelydid fossil recorded so far, only smaller than the relatively little-known *Sagulyda magna* Rasnitsyn, 1983, from the late Early or early Middle Jurassic of Central Asia.

In addition, the direction of 1-RS varies significantly; 1-RS is normally proclival (e.g., in *Prolyda*, *Xyelyda*, *Strophandria*, and *Ferganolyda*) or vertical (e.g., in *Sagulyda* and *Mesolyda*), resulting in the angles between 1-RS and 1-M ranging from 86.8° to 140° [3]. However, in the vast and extensive CNUB collection of more than 100 xyelydid fossils from China, *R. sticta* is the only one to date with 1-RS reclival. It also has 1-RS aligned linearly with 1-M (Figure 1G), thus forming a “T” shape, which is normally present in the Apocrita of the Hymenoptera [18,19]. Furthermore, the most basal sawflies usually possess SC in both wing pairs [1,2]. Xyelydidae, as the most basal and putative stem group (paraphyletic) in the Superfamily Pamphilioidea [4], have plesiomorphic characters with SC bifurcate in their forewings, but SC absent in the hind wings. *Rectilyda sticta,* however, has SC in both fore- and hind wings. In summary, unique and exceptional wing structures indicate that *R. sticta* has a combination of primitive and more derived characters highlighting its transitional role in the Xyelydidae.

As aforementioned, *Hoplitolyda duolunica* Gao, Shih, Rasnitsyn & Ren, 2013, collected from Sandaogou Township, Duolun County, is thus far considered to be the largest sawfly in the Praesiricidae, possibly in Symphyta [17]. Intriguingly, *R. sticta* is the second largest fossil specimen in the family Xyelydidae. It is unknown whether this phenomenon of gigantism is just a coincidence due to sampling bias from limited collection of fossil specimens or if it was caused by some factors in the ecosystem and environment at this locality. Gao et al. [17] discussed possible reasons why *Hoplitolyda duolunica* in the Mesozoic could have reached such a large size and stated that it might have been caused by food availability or sexual selection. For now, however, it is unclear if gigantism was a natural phenomenon at the Duolun locality and what factors could have resulted in giant body sizes, pending future collection of more large insect fossils from Duolun.

### About nygmata in the holometabola

Nygmata on the wings of various Holometabola have been known (often under different names) since at least the later part of the 19^th^ century. Martynov [20,21] made the first comprehensive study of nygmata using the term “facetic organs” to refer to McLahlan’s (1874–1880) “corneous points” as the first record that recognized these structures as very stable in their positions in respect to particular veins and forks in the wings of Trichoptera. Martynov demonstrated histologically that the “facetic organs” of Trichoptera were glandular in nature (he compared them with the wax glands of aphids and bees), which were most developed in the young adults (just molted), but degenerated in older adults. He also described the structures as being specific to Holometabola, but secondarily lost in Coleoptera, Diptera, and Lepidoptera. Of particular importance, he considered that numerous precursors found in Corydalidae (Megaloptera) indicate their possible abundance in the wings of ancestral Holometabola. However, studies and publications by Martynov [20,21] have not been broadly noticed, and even as recent as 1989 to 1991, nygmata have sometimes been referred to as “presumed sensory spots” [22,23]. However, the majority of authors recognize their glandular nature [24-26]. The idea of nygmata as a groundplan character of Holometabola has been recently reiterated by Minet et al. [27].

Nygmata are rarely present in compression fossils due to the nature of preservation. To date, nygmata have been reported on fossil wings in Permochoristidae, Permotanyderidae, Parachoristidae and Panorpidae of Mecoptera [4,28,29], Parasialidae of Megaloptera [30], Permithonidae of Neuroptera [29], Microptysmatidae of Trichoptera [29] and in Xyelidae of Hymenoptera (Figure 3B) [1,31]. In addition, Wang et al. recently reported nygmata in fossil neuropterans [32,33] and Liu et al. in fossil megalopterans [34].

### Nygmata evolution in hymenoptera

Of the extant Hymenoptera, nygmata are present only in Symphyta [35]. We examined various lower Apocrita (Orussidae, Stephanidae, Trigonalidae, Megalyridae and Gasteruptiidae s.l.) and were unable to find any nygmata (APR, pers. obs. 2014). Our observations, including the numbers and positions of nygmata for Symphyta, are summarized in Table 2. The data confirm that nygmata occur in almost every family of Symphyta, but not in every genus. Nygmata are particularly weakly visible, or apparently lost, in small sawflies, such as *Xyela* Dalman, *Pleroneura* Konow (Xyelidae), *Heterarthrus* Stephens (Tenthredinidae), etc. This may explain why nygmata are not detectable in *Syntexis* Rohwer, 1915, the only living representative of Anaxyelidae. On the other hand, there are sawflies of large body size without any visible nygmata, e.g., *Neodiprion sertifer* Geoffroy, 1785, Diprionidae.

**Table 2:** Summary of nygmata positions for extant Symphyta

	**1rm**	**2rm**	**3rm**	**1mcu**	**2mcu**	**1cua**	**1a**	**1rm**	**2+3rm**	**cua**	**1a**	**References**
**Xyelidae**												
*Macroxyela ferruginea *(Say, 1824)	+	+	-	-	-	-	+	-	+	+	-	http://www.morphbank.net/?id=102897http://www.morphbank.net/?id=102898
*Pleroneura coniferarum* (Hartig, 1837)	(+)	(+)	-	-	-	(+)	(+)	(+)	(+)	-	-	Orig.
*Xyela julii* (Dalman, 1820)	-	-	-	-	-	-	-	-	-	-	-	Orig.
**Blasticotomidae**												
*Blasticotoma filiceti* Klug, 1834	+	+	+						+		+	Orig.
**Argidae**												
*Arge ustulata* (Linné, 1758)	-	+	-	-	-	-	-	-	-	-	-	Orig.
*Aprosthema* sp.	-	-	-	-	-	-	-	-	-	-	-	Orig.
*Scobina stigmaticollis* (Klug, 1834)	+	+	-	-	-	-	-	-	-	-	-	Orig.
*Pachylota audouinii* Westwood, 1841	-	+	-	-	-	-	-	-	-	-	-	Orig.
**Pergidae**												
*Perga affinis* Kirby, 1882	+	+	-	-	-	-	-	-	-	-	-	Orig.
*Lophyrotoma interrupta* (Klug, 1814)	+	+	-	-	-	-	-	-	-	-	-	Orig.
*Phylacteophaga eucalypti* Froggatt, 1899	-	-	-	-	-	-	-	-	-	-	-	Orig.
**Cimbicidae**												
*Cimbex connatus* (Schrank, 1776)	-	-	-	-	-	-	-	-	-	-	-	Orig.
*Zarea fasciata*	-	-	-	-	-	-	-	-	-	-	-	Orig.
*Pachylosticta albiventris* Klug, 1824	-	-	-	-	-	-	-	-	-	-	-	Orig.
*Corynus amoena* (Klug, 1834)	-	-	-	-	-	-	-	-	-	-	-	Orig.
*Corynus obscura* (Fabricius, 1775)	-	-	-	-	-	-	-	-	-	-	-	Orig.
**Diprionidae**												
*Neodiprion sertifer* (Geoffroy, 1785)	-	-	-	-	-	-	-	-	-	-	-	Orig.
**Tenthredinidae**												
*Croesus latipes* (Villaret, 1832)	+	+	-	-	-	-	-	-	+	-	-	Orig.
*Pachynematus* sp.	+	+	-	-	-	-	-	-	+	-	-	Orig.
*Hemichroa australis* (Serville, 1823)	+	+	-	-	-	-	-	-	+	-	-	Orig.
*Selandria* sp.	-	+	-	-	-	-	-	-	+	-	-	Orig.
*Dolerus vestigialis* (Klug, 1818)	-	-	-	-	-	-	-	-	-	-	-	Orig.
*Dolerus gonager* (Fabricius, 1781)	-	-	-	-	-	-	-	-	-	-	-	Orig.
*Eriocampa* sp.	+	+	+	-	-	-	-	-	-	-	-	Orig.
*Sciapterix consobrina* (Klug, 1816)	+	+	-	-	-	-	-	-	-	-	-	Orig.
*Empria candidata* (Fallén, 1808)	+	+	-	-	-	-	-	-	-	-	-	http://www.morphbank.net/?id=716093
*Empria takeuchii* Prous, Heidemaa, 2011	-	+	-	-	-	-	-	-	-	-	-	http://www.morphbank.net/?id=693606
*Empria formosana* Prous, Heidemaa, 2012	+	+	-	-	-	-	-	-	-	-	-	http://www.morphbank.net/bischen/?id=786403
*Blennallantus compressicornis* Wei, 1998	(+)	(+)	-	-	-	-	-	-	-	-	-	http://www.morphbank.net/?id=716091http://www.morphbank.net/?id=716092
*Tenthredo bifasciata rossii* (Panzer, 1803)	+	+	+	-	-	-	-	-	+	+	-	Orig.
*Hetererthrus vagans* (Fallen, 1808)	-	-	-	-	-	-	-	-	-	-	-	Orig.
*Athalia rosae* (Linné, 1758)	+	+	-	-	-	-	-	-	-	-	+	Orig., http://www.morphbank.net/?id=102498http://www.morphbank.net/?id=102497
**Pamphiliidae**												
*Cephalcia abietis* (Linné, 1758)	+	+	+	-	+	-	+	-	+	+	+	Orig.
*Acantholyda erythrocephala* (Linné, 1758)	+	+	+	-	-	+	+	+	+	+	+	Orig.
*Pamphilius pallipes* Zetterstedt, 1838	+	+	+	-	-	-	+	-	+	+	+	Orig.
*Onycholyda amplecta* (Fabricius, 1804)	+	+	+	+	-	-	+		+	(+)	(+)	http://www.morphbank.net/?id=102851http://www.morphbank.net/?id=102852
**Megalodontesidae**												
*Megalodontes* sp.	+	+	+	-	-	-	-	-	-	-	-	Orig.
**Cephidae**												
*Syrista parreyssii* (Spinola, 1843)	-	+	-	-	-	-	-	-	-	-	-	Orig.
*Calameuta pallipes* (Klug, 1803)	-	-	-	-	-	-	-	-	-	-	-	Orig.
*Cephus pygmeus* (Linné, 1767)		+		-	-	-	-	-	-	-	-	http://www.morphbank.net/?id=102959http://www.morphbank.net/?id=102960
**Anaxyelidae**												
*Syntexis libocedrii* Rohwer, 1915	-	-	-	-	-	-	-	-	-	-	-	Orig.
**Siricidae**												
*Urocerus gigas* (Linné, 1758)	+	+	+		2	+	-	-	2	+	-	Orig.
*Sirex noctilio* Fabricius, 1793	+	+	+		2	+	-	-	2	-	-	Orig.
*Tremex fuscicornis* (Fabricius, 1787)	+	+	+	-	+	+	-		2	+	-	Orig.
*Tremex columba* (Linné, 1763)	+	+	+	-	-	+	-	-	+	-	-	http://www.morphbank.net/?id=102782http://www.morphbank.net/?id=102763
**Xiphydriidae**												
*Xiphydria camelus* (Linné, 1758)	+	+	-	-	-	-	-		+			Orig., http://www.morphbank.net/?id=102720http://www.morphbank.net/?id=102721
*Euxiphydria potanini* (Jakowlew, 1891)	+	+	-	-	-	-			+			Orig.
**Orussidae** spp.	-	-	-	-	-	-	-		-			Orig.

(+) nygma weakly developed.

Generally, the families with the highest number of cells with nygmata are in the extant Pamphiliidae (up to five in the forewing and four in the hind wing) and Siricidae (up to five in the forewing and only two in the hind wing). The lowest number of cells with nygmata is recorded in the Cephidae (one and zero for the fore- and hind wing, respectively). Other families take intermediate positions. Even the most basal hymenopteran family, Xyelidae, shows a modest development of nygmata (up to three and two for the fore- and hind wing, respectively), unless considering the four and two rudimentary nygmata in *Pleroneura* Konow, 1897. The nygmata of Symphyta are characteristically found in the forewing cells of 1rm and 2rm and in the hind wing cell of 2 + 3rm. Nygmata in the forewing 2rm and hind wing 2 + 3rm cells are the most stable. Minet et al. [27] have argued that nygmata located in the RS-M interspace are groundplan autapomorphies of the entire Holometabola (referring to Xyelidae as an example in Hymenoptera). In the case of venational reduction, the aforementioned stable set of nygmata would be useful for exploring cell and vein homology, provided the nygmata are persistent.

**Figure 4:**
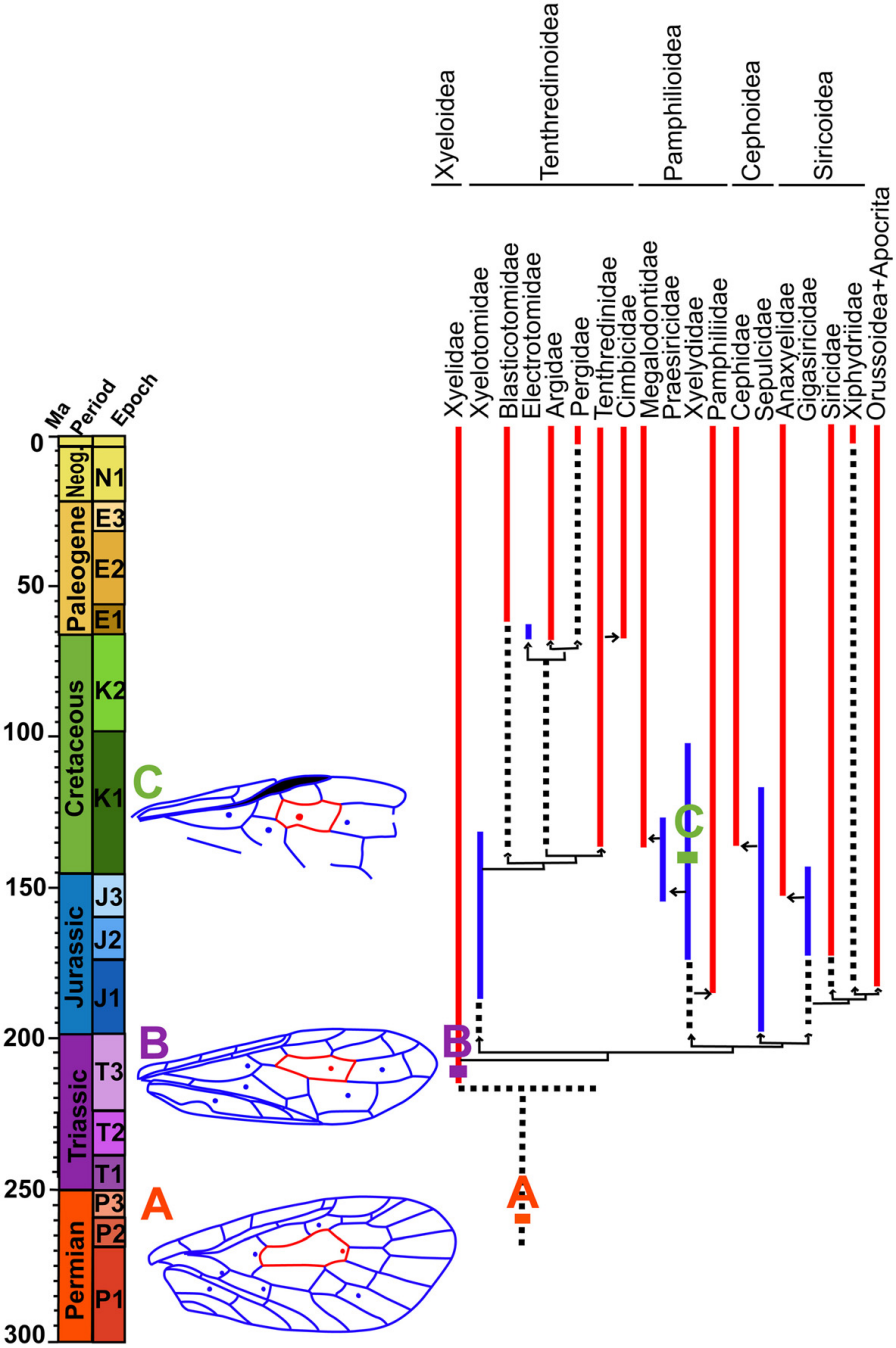
**Noncladistic cladogram of basal Hymenoptera after Rasnitsyn (2002, fig 331, partial, modified), with forewing venation containing nygmata among fossil taxa mapped onto figure. A**, *Parasialis latipennis* Ponomarenko, 1977 (Parasialidae). **B**, Xyelidae (based on *Asioxyela paurura* Rasnitsyn, 1969 and *Madygenius primitivus* Rasnitsyn, 1969; after Shcherbakov, 2013). **C**, *Rectilyda sticta* gen. et sp. nov. (Xyelydidae).

Of the fossil Xyelidae, both Triassic *Asioxyela paurura* Rasnitsyn, 1969, and *Madygenius primitives* Rasnitsyn, 1969, have six nygmata in forewing cells 1rm, 1cua, 1a, 2rm, 3rm, and 2mcu [1,31] (Figure 4B). This implies that the low number of nygmata in the living Xyelidae (Table 2) is the result of a secondary reduction. In fact, Xyelidae, the most basal hymenopteran family, is the most plesiomorphic in that respect as well. *Rectilyda sticta* gen. et sp. nov. in the family of Xyelydidae, the only other sawfly fossil with nygmata preserved, has four symmetrical nygmata on each forewing (in cells 1rm, 2rm, 3rm and 1mcu) and two in the hind wing, both in the cell 2 + 3rm (Figure 4C). The latter two nygmata in one cell of the hind wing probably pertain to an origin as isolated cells of 2rm and 3rm, as is still retained by many members of the Xyelidae. The siricid forewings are analogous; their cells 2rm and 3rm each preserve a nygma, even if a delimiting crossvein 2r-m is lost (as in *Tremex* Jurine, 1807).

The family Xyelidae appeared in the Middle or Upper Triassic of Kyrgyzstan in Central Asia [1,2] and the Upper Triassic of Australia [36,37], South Africa [38] and Argentina [39]. It is considered the most basal group in the phylogeny of Symphyta and in Hymenoptera [2,4,7,40,41]. Shcherbakov [31,42] considers Permian Parasialidae Ponomarenko, 1977 (Figure 4A) [Suborder Archimegaloptera Engel, 2004, Order Panmegaloptera Shcherbakov, 2013 (=Megaloptera s.l., i.e. *sensu* Latreille, 1802)] to be ancestors of Hymenoptera, because they are similar to symphytans in venation, pterostigma, and nygmata. However, this assumption contradicts several characters implying Hymenoptera to be a sister group of all the remaining Holometabola. In particular, the ovipositor structure of other Holometabola have synapomorphies in having the second valves (gonapophyses of the 9^th^ segment or dorsal stylets) reduced, and the third valves (outer valves, derivatives of valvifers 2) fused and working as an intromittent organ inserted into the substrate during oviposition. In contrast, the groundplan hymenopteran ovipositor retains a pterygotan ground plan in that its 2^nd^ valves are developed and functional, and the 3^rd^ valves are free while serving as saw-like sheaths. Additionally, current molecular phylogenies mainly show that Hymenoptera is a sister group of the remaining Holometabola [41,43-48]. Nevertheless, wings of the Parasialidae (Figure 4A) could be taken as a rough model of a hymenopteran ancestor until a more likely ancestral group, the extinct order Palaeomanteida [2,49], is studied for nygmata in the future.

There is a general trend of a decreasing number of nygmata during the evolution of hymenopteran wings. This is evidenced by the seven forewing nygmata of *Parasialis latipennis* Ponomarenko, 1977 in Parasialidae in the Permian (Figure 4A), six nygmata for *Asioxyela paurura* and *Madygenius primitives* in Xyelidae in the Triassic [1,31] (Figure 4B), and one to three nygmata in most of the extant species among Symphyta (Figure 3A-E, G-J), except for Pamphiliidae and Siricidae, which have five (Figure 3F). *Rectilyda sticta* gen.et sp. nov., with its four nygmata, appears to have a transitional role in this general trend.

## Conclusions

A thorough review of the various types of nygmata in extant and extinct Hymenoptera suggests that in this order, the nygmata have had stable positions in the forewing cell of 2rm and hind wing cell of 2 + 3rm. In addition, the evolution of the Hymenoptera has generally resulted in a decrease in the number of nygmata. Most importantly, *R. sticta* gen. et sp. nov., with four nygmata, bridges the gap and provides rare insights into the trend of nygmata development over time, as well as the evolution of insects in general and of hymenopterans in particular.

### Availability of supporting data

The data set supporting the results of this article is available in the Dryad repository, doi:10.5061/dryad.v561f [50].

### Ethics

The authors declare that the study makes no uses of human, clinical tools and procedures, vertebrate and regulated invertebrate animal subjects and/or tissue, and plants.

## Competing interests

The authors declare that there are non-financial competing interests (political, personal, religious, ideological, academic, intellectual, commercial or any other), no competing interests in the manuscript.

## Authors’ contributions

MW, APR, CKS carried out the fossil processing, photography and figure preparation. MW, CKS, DR conceived and designed the study. MW, APR, CKS, DR participated in the data analysis, interpretation, manuscript drafting, modification and finalization. All authors read and approved the final manuscript.
